# Bacterial Exposure to Nickel: Influence on Adhesion and Biofilm Formation on Orthodontic Archwires and Sensitivity to Antimicrobial Agents

**DOI:** 10.3390/ma14164603

**Published:** 2021-08-16

**Authors:** Andrej Pavlic, Gabrijela Begic, Marin Tota, Maja Abram, Stjepan Spalj, Ivana Gobin

**Affiliations:** 1 Department of Orthodontics, Faculty of Dental Medicine, University of Rijeka, 51000 Rijeka, Croatia; pavlic.andrej@yahoo.com; 2 Department of Pediatric Dentistry, Faculty of Dental Medicine, University of Rijeka, 51000 Rijeka, Croatia; 3 Department of Microbiology and Parasitology, Faculty of Medicine, University of Rijeka, 51000 Rijeka, Croatia; gabrijela.begic@medri.uniri.hr (G.B.); maja.abram@medri.uniri.hr (M.A.); ivana.gobin@medri.uniri.hr (I.G.); 4 Department of Medical Chemistry, Biochemistry and Clinical Chemistry, Faculty of Medicine, University of Rijeka, 51000 Rijeka, Croatia; marin.tota@medri.uniri.hr; 5 Department of Dental Medicine, Faculty of Dental Medicine and Health, J.J. Strossmayer University of Osijek, 31000 Osijek, Croatia

**Keywords:** bacteria, nickel, biofilm, adhesion, orthodontic archwire, antimicrobial resistance

## Abstract

The presence of nickel could modify bacterial behavior and susceptibility to antimicrobial agents. Adhesion and biofilm formation on orthodontic archwires can be a source of bacterial colonization and possible health hazards. *Staphylococcus aureus* was subjected to exposure and adaptation to various sub-inhibitory concentrations of nickel. Five strains of bacteria adapted to nickel in concentrations of 62.5–1000 μg/mL were tested for adhesion and biofilm formation on nickel-titanium archwires. Archwires were previously incubated in artificial saliva. Bacteria were incubated with orthodontic wire with stirring for 4 h (adhesion) and 24 h (biofilm formation). The number of adherent bacteria was determined after sonication and cultivation on the Muller-Hinton agar. Disk diffusion method was performed on all bacteria to assess the differences in antimicrobial susceptibility. Bacteria adapted to lower concentrations of nickel adhered better to nickel-titanium than strains adapted to higher concentrations of nickel (*p* < 0.05). Biofilm formation was highest in strains adapted to 250 and 500 μg/mL of nickel (*p* < 0.05). The highest biofilm biomass was measured for strains adapted to 250 μg/mL, followed by those adapted to 1000 μg/mL. Bacteria adapted to lower concentrations of nickel demonstrated lower inhibition zone diameters in the disk diffusion method (*p* < 0.05), indicating increased antimicrobial resistance. In conclusion, bacteria adapted to 250 μg/mL of nickel ions adhered better, demonstrated higher biofilm formation and often had higher antimicrobial resistance than other adapted and non-adapted strains.

## 1. Introduction

Nickel can be released to the human body by oral corrosion of nickel-containing dental alloys used for dental instruments, restorations and orthodontic appliances [1]. Orthodontic treatment with fixed appliances lasts about 2–2.5 years, and numerous studies have shown corrosion and release of nickel into the oral cavity [2]. Nickel-titanium (NiTi) archwires are widely used because of their desirable mechanical properties, although they can be a significant contributor to the corrosion of nickel ions. On average, 40 μg of nickel are released daily from a fixed orthodontic appliance [3], with the highest release in the first week and significant deceleration over time [4,5]. The release of nickel from orthodontic appliances is lower in static conditions than under functional stress, but the additional dietary intake of nickel ranging from 130–165 μg per day and the cumulative effect during long-lasting treatment should also be considered [6]. Our data on the dynamics of nickel release from orthodontic NiTi archwires during a 28-day exposure to saliva have been previously reported [7]. Briefly, the greatest release of nickel is in the first three days (0.3 μg/cm^2^ of the wire surface), followed by a decreasing tendency, implying the formation of the protective oxide film. The cumulative release over 28 days amounted to 0.7 μg/cm^2^ of the wire surface. There is some evidence pointing out that elevated nickel levels are found in tissues of patients who have had an orthodontic appliance for one year [8]. Nickel has been shown to have various effects on bacteria. In lower concentrations, it is used as a micronutrient, but in higher concentrations can be bacteriostatic [9,10]. This phenomenon is gaining more interest in the bacterial cross-adaptation and resistance to antibiotics field, which is induced by exposure to heavy metals. The mechanisms of adaptation and resistance of bacteria to heavy metal can be achieved by reduced membrane permeability, activation of the efflux pump, inactivation or mutation of gene encoding targets of both antibiotics and metals and by biofilm formation [11,12,13,14].

Standardized techniques for testing antibacterial effects are based on either diffusion (e.g., Kirby-Bauer test), dilution (determination of the minimal inhibitory concentration (MIC)) or a combination of diffusion and dilution (E-test). A common feature of standard methods is the endpoint or regular-interval growth determination [15]. A disadvantage of these methods is that the dynamics of bacterial growth cannot be monitored with a combination of different substances, like metals and antibiotics. It is also well known that pH, temperature and organic matter content can affect bacterial growth, which can be examined by monitoring the bacterial growth curve [16].

Increased adhesion of bacteria on elements of the orthodontic appliance could lead to a formation of biofilm and an increased number of pathogenic bacteria in the oral cavity, with several factors determining the amount [17]. This can create malfunctioning of some orthodontic elements such as elastomeric chains [18], but more importantly, various health hazards [19].

The microbial biofilm is a complex structure made of prokaryotic and/or eukaryotic cells embedded in a matrix composed of materials synthesized by microbial communities that grow on various structures such as various abiotic surfaces, human tissues etc. [20,21].

*S. aureus* is a Gram-positive facultative anaerobe and a human organism commensal [22] which can be responsible for invasive, life-threatening infections, such as bacteremia, infective endocarditis, toxic shock syndrome, osteomyelitis, and others. In addition, numerous strains of this bacterium demonstrate increased antimicrobial resistance [23]. *S. aureus* is more frequently present in the oral cavity than was previously expected. Furthermore, it has been linked with various oral diseases such as cheilitis, parotitis and mucositis [24,25].

The aim of this study was to isolate the bacteria that adapted to various concentrations of nickel and to assess the effect of nickel ions on the adhesion and early biofilm formation of those bacteria on NiTi orthodontic archwires. Furthermore, the strains of *S. aureus* adapted to nickel were subjected to a disk diffusion and growth curve test to determine potential changes in antibiotic susceptibility.

The hypotheses were:

- bacteria adapted to lower concentrations of nickel ions will adhere better to NiTi wires;
- bacteria adapted to lower concentrations of nickel ions will demonstrate better biofilm formation to NiTi wires;
- bacteria adapted to lower concentrations will show a decreased sensitivity to antimicrobial agents.

## 2. Materials and Methods

### 2.1. Strain and Growth Media

The *Staphylococcus aureus* strain American Type Cell Culture (ATCC, Microbiologics, St. Cloud, MN, USA) 29,213 was used. The bacteria were kept frozen at −80 °C after being dispensed with an addition of 10% glycerol. For each experiment, an aliquot was thawed and grown in Mueller-Hinton (MH) broth (Biolife Italiana, Milano, Italy) for 24 h and then subcultured on an MH agar (Biolife Italiana, Milano, Italy). The concentration of bacteria was determined by measuring optical density (OD) on spectrophotometer Biofotometer (Eppendorf, Hamburg, Germany) at 600 nm. The OD of 1 set at 600 nm corresponds to approximately 1 × 10^9^ colony-forming units per milliliter (CFU/mL). Three series of tenfold dilutions were made to set the bacterial suspension at 10^6^ CFU/mL. Bacterial inoculum were later verified by diluting and plating the dilutions onto MH agar and incubated for 24 h.

### 2.2. Preparation of Reagents

All reagents used were of analytical and molecular biology grade. A stock solution of NiCl_2_ *×* 6H_2_O (Sigma-Aldrich, St. Louis, MO, USA) was prepared in double-distilled water, adjusted to pH of 7, passed through a 0.45 µm syringe filter and stored at 4 °C. The stock concentration was 8000 μg/mL. The working concentration of NiCl_2_ *×* 6H_2_O solution was prepared by suitable dilutions of the stock solution.

### 2.3. Adaptation of S. aureus to Nickel

The concentrations of nickel solution that were used in the adaptation process were, from 1st to 5th: 62.5, 125, 250, 500 and 1000 μg/mL. Such a broad range was used to take into account the cumulative nickel concentrations during long-term orthodontic treatment, the higher release at lower pH of saliva and biofilm and the dietary intake of nickel. The NiCl solutions used in this experiment were used as blank samples to avoid affecting the bacterial growth readings. Firstly, the bacteria were incubated at 37 °C for 24 h in a broth containing the sub-inhibitory concentration of nickel (62.5 μg/mL). Afterward, it was transferred to a broth containing a twofold concentration (125 μg/mL) of nickel incubated at 37 °C for 24 h. Simultaneously, the incubated bacteria were grown on an MH agar containing the same concentration of nickel as in MH broth and incubated for another 24 h, and then collected and stored in a glycerol broth at −80 °C. Following the same pattern, the bacteria were incubated and stored up to the concentration where no visible bacterial growth was detected. The selected bacteria were grown in a medium without nickel for 2–3 days, and afterward, returned to the concentration of nickel from which they were isolated. Positive growth response was considered an adaptation to nickel.

### 2.4. Minimal Inhibitory Concentrations and Disk Diffusion

A resazurin-based microdilution method was used to determine the minimum inhibitory concentrations (MIC) of nickel for *S. aureus* ATCC. Serial twofold dilutions of reagents ranging from 2000 to 125 μg/mL were made in a microtiter plate (Vacutest Kima, Arzergrande, Italy). In each well, a bacterial suspension (1 × 10^6^ CFU/mL per well) and the resazurin (0.015% solution) (Sigma-Aldrich, St. Louis, MO, USA) were added. Three wells were used as sterility controls and three as growth controls. After 24 h of incubation at 37 °C, plates were read visually. The lowest concentration that did not show a change in color (blue) was defined as MIC.

Disk diffusion method was performed on all bacteria to assess the differences in antimicrobial susceptibility. The method was done according to the European Committee on Antimicrobial Susceptibility Testing (EUCAST) protocol [26]. It was performed in triplicate, and the readings were done by the same laboratory technician.

The following antimicrobial agents and concentrations were used (average inhibition zone diameter in mm according to EUCAST is shown in parenthesis):

- Benzylpenicillin—P—1 unit (12–18)
- Clindamycin—CMN—2 µg (23–29)
- Erythromycin—ERY—15 µg (23–29)
- Gentamicin—GMN—10 µg (19–25)
- Cefoxitin—FOX—30 µg (24–30)
- Teicoplanin—TEC—30 µg (10–14)
- Linezolid—LIN—10 µg (21–27)
- Ciprofloxacin—CIP—5 µg (21–27)
- Rifampicin—RIF—5 µg (30–36)
- Trimethoprim-sulfamethoxazole—SXT—1.25–23.75 µg (26–32)
- Moxifloxacin—MXF—5 µg (25–31)

Before the adhesion tests, the adapted, frozen bacteria were defrosted and underwent a novel incubation in a broth with the first five nickel concentrations.

### 2.5. Growth Curve Assays

Microplates with three chosen strains were prepared with various concentrations of one chosen antimicrobial agent—ciprofloxacin (Sigma-Aldrich, St. Louis, MO, USA) and incubated in the microplate reader (Microplate Reader ELX 808, Biotek, Winooski, VT, USA) for 24 h in order to obtain growth curves with measurements of OD every hour. Measures were performed in triplicate. Growth curves of bacteria not adapted to nickel were compared to the ones adapted to concentrations of nickel of 250 μg/mL and 1000 μg/mL.

### 2.6. Adhesion, Early Biofilm and Biofilm Biomass Tests

Adhesion and biofilm tests were performed on 0.018 × 0.025 inch NiTi archwires (Dentsply GAC, York, PA, USA). Orthodontic archwires were previously incubated overnight in artificial saliva. Saliva was composed of: mucin (0.25 weight/volume (*w*/*v*)), sodium chloride (0.35 *w*/*v*), potassium chloride (0.02 *w*/*v*), calcium chloride dihydrate (0.02 *w*/*v*), yeast extract (0.2 *w*/*v*), laboratory lemco powder (0.1 *w*/*v*) Oxoid—bovine extract, protease peptone (0.5 *w*/*v*), distilled water and urea [27]. Bacteria were transferred to wells with orthodontic wire and stirred for 4 h (adhesion) and 24 h (biofilm formation). At the end of the incubation period, the wires were subjected to double rinsing with saline solution and sonicated for 1 min at an intensity of 40 kHz. After homogenization, ten-fold dilutions were made in microtiter plates, and the bacteria were plated and incubated at 37 °C. Crystal violet staining was used to determine total biofilm biomass on orthodontic archwires [28]. Biomass testing was performed for *S. aureus* ATCC, and strains were adapted to concentrations of nickel of 250 μg/mL and 1000 μg/mL.

All experiments were repeated three times in duplicate.

### 2.7. Statistics

The data were analyzed with IBM SPSS software (Version 22, 2013, IBM Corp, Armonk, NY, USA). Bacterial counts are log-transformed to get a more normal distribution. Student’s *t*-test for independent samples, paired-samples *t*-test, one-way analysis of variance (ANOVA) and Student-Newman-Keuls post-hoc were used for statistical analyses. The effect size was quantified by η^2^ for ANOVAs, and for *t*-test by using the formula r = √ (t^2^/(t^2^ + df)). Cohen’s criteria were used in the interpretation of r: 0.1–0.3 = small effect size, 0.3–0.5 = moderate, 0.5–0.7 = large, and > 0.7 very large. The r^2^ values were used to interpret η^2^.

### 2.8. Ethics

The study was approved by the Institutional Ethics Committee Review Board of the University of Rijeka Faculty of Medicine (No. 2170-24-04-3-18-5; 19/12/2018).

## 3. Results

### 3.1. Adhesion, Early Biofilm and Biomass Tests

**Figure 1:**
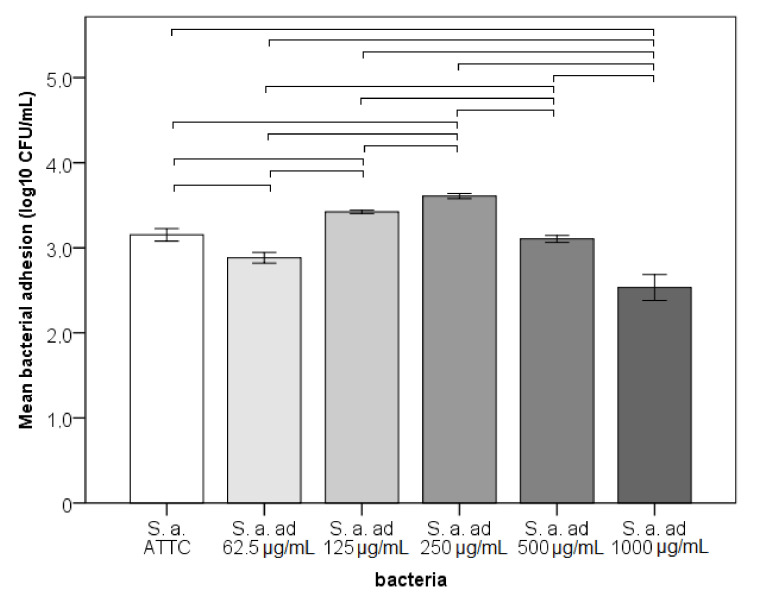
Adhesion of bacteria to NiTi archwires—bacterial count in CFU/mL (ad—adapted to Ni^2+^, horizontal line connect strains that differ significantly).

Adhesion on NiTi significantly differed between strains with a very large effect size (*p* < 0.001; η^2^ = 0.958; Figure 1). Strains adapted to 62.5 μg/mL Ni^2+^ and 1000 μg/mL had significantly lower adhesion than ATCC strain, while 125 μg/mL and 250 μg/mL significantly higher (*p* < 0.05). *S. aureus* adapted to 250 μg/mL demonstrated the highest adhesion over the others, while the strain adapted to 1000 μg/mL had the lowest. The strain adapted to 500 μg/mL had similar adhesion as the ATCC strain.

**Figure 2:**
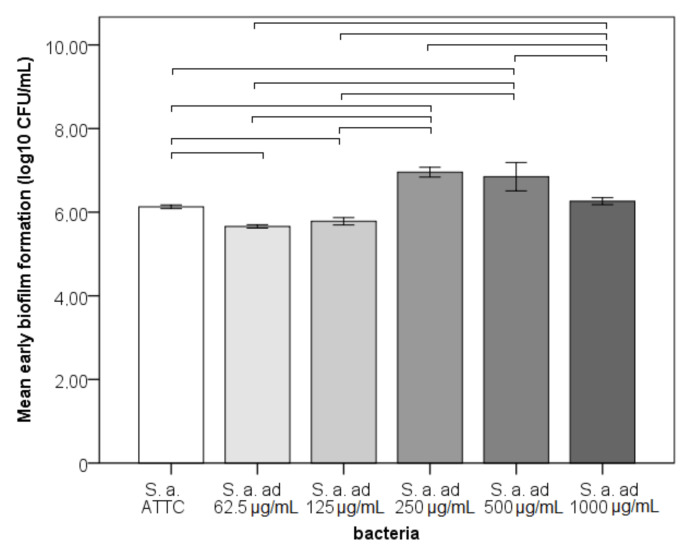
Early biofilm formation of bacteria to NiTi archwires—bacterial count in CFU/mL (ad—adapted Ni^2+^, horizontal line connect strains that differ significantly).

Early biofilm formation on NiTi significantly differed between strains with a very large effect size (*p* < 0.001; η^2^ = 0.916; Figure 2). Strains adapted to 250 and 500 μg/mL demonstrated the highest biofilm formation among the strains (*p* < 0.05). The ATCC strain biofilm formation was not different from the 1000 μg/mL adapted strain, was lower than the 250 and 500 μg/mL adapted strains and was higher than the 62.5 and 125 μg/mL adapted strains.

**Figure 3:**
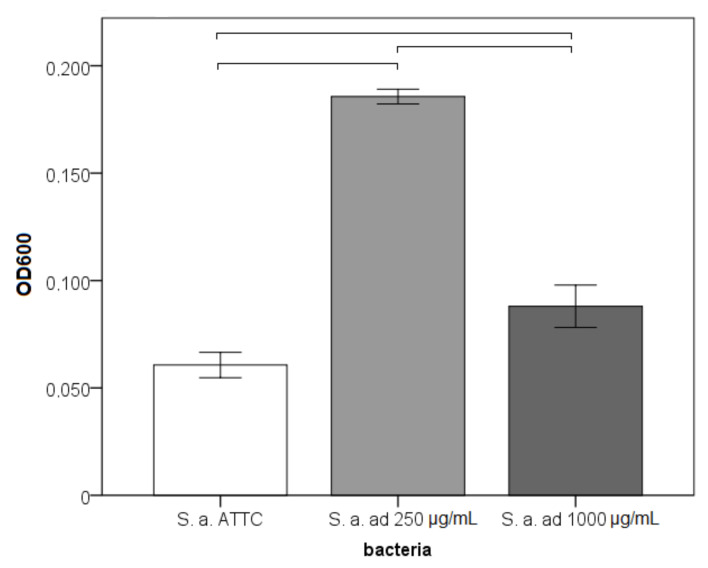
Biofilm biomass of bacteria on nickel-titanium archwires (OD600—optical density measured at 600 nm, ad—adapted to Ni^2+^, horizontal line connect strains that differ significantly).

The total biofilm biomass was significantly different between the adhered *S. aureus* ATTC and the strains adapted to 250 and 1000 μg/mL with very large effect sizes (*p* < 0.001; η^2^ = 0.986). The highest biomass was measured for the strain adapted to 250 μg/mL, followed by the strain adapted to 1000 μg/mL (Figure 3).

### 3.2. Growth Curve Assays

**Figure 4:**
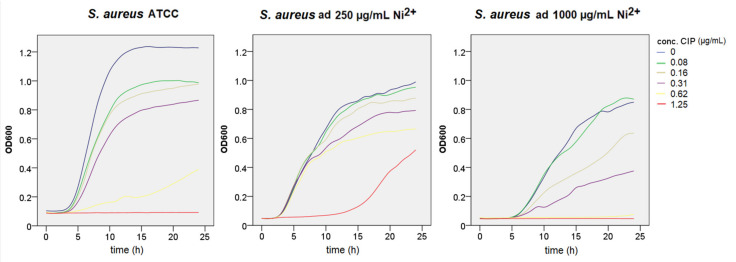
Growth curves of *S. aureus* strains in different ciprofloxacin concentrations measured at an optical density of 600 nm (Ni—nickel, CIP—ciprofloxacin).

MIC for ciprofloxacin for *S. aureus* ATCC was 0.62 μg/mL. *S. aureus* adapted to 250 μg/mL demonstrated better growth in bacteriostatic and bactericidal concentrations of ciprofloxacin. Strain adapted to 1000 μg/mL had inhibited growth in ciprofloxacin concentrations when compared to the ATCC strain (Figure 4).

**Figure 5:**
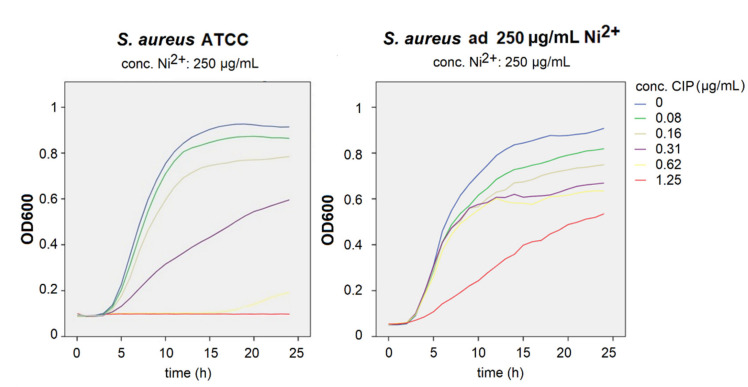
Growth curves of *S. aureus* strains of ATCC in 250 μg/mL of nickel and adapted to that concentration of nickel in different ciprofloxacin concentrations measured at an optical density of 600 nm (Ni—nickel, CIP—ciprofloxacin).

**Figure 6:**
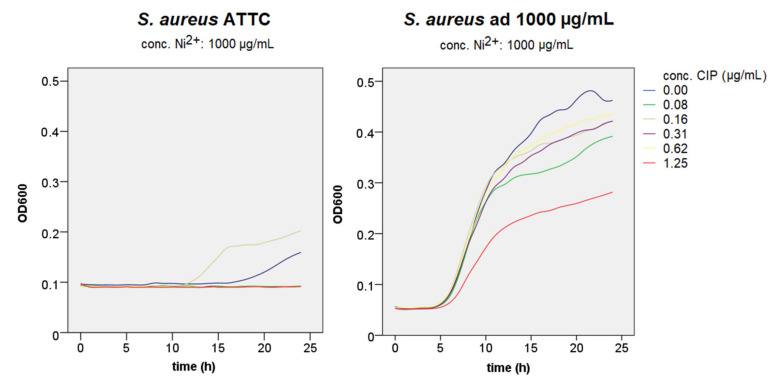
Growth curves of *S. aureus* strains ATCC in 1000 μg/mL nickel and adapted to that concentration of nickel in different ciprofloxacin concentrations measured at an optical density of 600 nm (Ni—nickel, CIP—ciprofloxacin).

A comparison of growth curves for *S. aureus* ATCC with the addition of 250 μg/mL of nickel and bacteria adapted to that nickel concentration showed that even short-term adaptation to nickel could lead to antimicrobial resistance (Figure 5). It could be seen that adapted bacteria grew even with a higher concentration of antibiotics (purple and red curve). A similar pattern was seen by comparing ATCC with the addition of 1000 μg/mL of nickel and bacteria adapted to that same concentration (Figure 6). The ATCC bacteria did not grow on that concentration, while the adapted one did grow, but at a slower rate.

### 3.3. Antimicrobial Susceptibility

**Figure 7:**
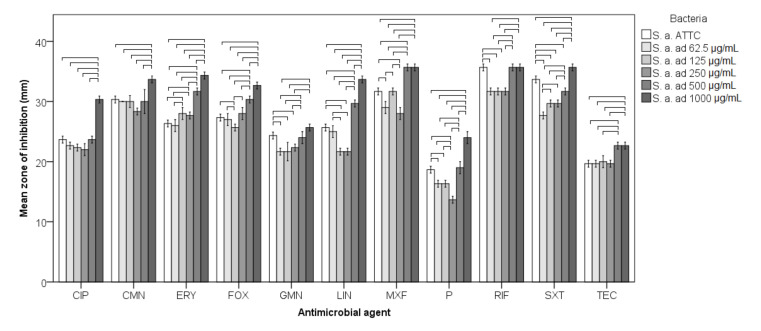
Antimicrobial sensitivity of adapted and non-adapted bacteria evaluated by disk diffusion method expressed in diameter of inhibition. S. a.—*Staphylococcus aureus*; ad—adapted to Ni^2+^, CIP—Ciprofloxacin; CMN—Clindamycin; ERY—Erythromycin; GMN—Gentamicin; FOX—Cefoxitin; LIN—Linezolid; MXF—Moxifloxacin; P—Benzylpenicillin; RIF—Rifampicin; SXT—Trimethoprim-sulfamethoxazole; TEC—Teicoplanin; horizontal line connect strains that differ significantly.

MIC for nickel was 1000 μg/mL. Disk diffusion results showed that all tested strains, including the ATCC and adapted strains, were sensitive to tested antimicrobial agents according to the EUCAST values for disk diffusion. However, some tendencies towards higher and lower sensitivity to antibiotics were observed when differences in diameter values of disk diffusion results were analyzed. Differences in antimicrobial susceptibility between strains for every antimicrobial agent were significant with very large effect sizes (*p* < 0.001; η^2^ = 0.794–0.984; Figure 7).

*S. aureus* adapted to 500 μg/mL of Ni^2+^ demonstrated significantly higher sensitivity to a series of antibiotics (CIP, CMN, ERY, FOX, LIN, MXF, P, SXT, TEC) compared to the ATCC strain (*p* < 0.05) and so did *S. aureus* adapted to 250 μg/mL of Ni^2+^ (to ERY, FOX, LIN, MXF, TEC) (*p* < 0.05). Bacteria adapted to 250 μg/mL were significantly more resistant only to SXT (*p* < 0.05). On the other hand, bacteria adapted to 62.5–250 μg/mL were significantly more resistant to some antibiotics when compared to the ATCC strain, adapted to 62.5 μg/mL to GMN, P, RIF, SXT, MXF, adapted to 125 μg/mL to FOX, GMN, LIN, P, RIF, SXT and adapted to 250 μg/mL to GMN, LIN, MXF, P, RIF, SXT (*p* < 0.05) (Figure 7).

## 4. Discussion

This research confirmed that corrosion-induced nickel release from orthodontic appliances could modify the behavior of bacteria in the oral cavity depending on the dose of released nickel. Exposure to low nickel concentrations appears to have a low potential to alter bacterial adherence and biofilm formation but greater potential to induce bacterial resistance to antimicrobial agents. On the other hand, bacteria exposed to higher nickel concentrations tend to be more susceptible to antimicrobial agents and display reduced adhesion to orthodontic NiTi archwires.

In addition, the dose does not have the same effect on adhesion and early biofilm formation. Exposure to low (62.5 and 125 μg/mL) and high doses (1000 μg/mL) does not affect the adhesion and formation of early biofilm much, although low doses tend to reduce adhesion. As the biofilm matures, there is no difference in the number of bacteria because the bacteria behave differently within the biofilm.

It seems that the exposure and adaptation to moderate nickel concentrations enabled the bacteria to adhere better on orthodontic NiTi archwires and form a biofilm when compared to the control strain. Bacteria adapted to 250 μg/mL of nickel showed the highest adhesion potential, biofilm accumulation and biofilm mass. Moderate concentrations of nickel are likely to deplete the defense system, alter the polarity of the membrane and increase cell hydrophilicity, increasing adhesion to saliva-coated wires and binding to the biofilm as the bacterium tries to increase its chance of survival.

Bacteria evolved mechanisms of metal tolerance to avoid cellular damage caused by metal ions. Formation or sequestration of toxic metals, reduction of intracellular ions for detoxification and extrusion of toxic ions by efflux systems are the three main mechanisms of heavy metal resistance [29,30,31].

Research has shown that bacteria accumulate nickel [32]. Biosorption of toxic metals has been demonstrated in bacterial cell membranes, cell walls and extracellular polymeric substances (EPS) of biofilms [33]. Keeping in mind that the daily release of nickel solely from NiTi archwires can elevate up to 40 µg per day [3], the finding could be a potential clinical issue. Modification of conditions in the oral cavity, such as the use of agents for plaque control like fluorides and chlorhexidine [34,35], can further increase the corrosion of NiTi wires [36]. *Staphylococcus aureus* presence in the oral cavity is underestimated, and its modification by nickel ions and adhesion on orthodontic appliances could pose a problem [37]. Furthermore, the risk from the dissemination of *S. aureus* to other areas increases with its habitation in the mouth [38]. One solution may be coating NiTi wires, which seem to have a lower adhesion potential [39].

Strain adapted to a high concentration of nickel of 1000 μg/mL showed less adhesion potential in comparison to the ATCC strain, but higher biofilm biomass was measured. Excessive concentrations of nickel likely stimulate bacteria to produce a lot of EPS in order to protect themselves, resulting in higher biomass. It has been reported that the EPS matrix and the contained polysaccharides bind heavy metals [40]. Biofilm adhesion is a potential risk for orthodontic treatment with the fixed appliance, as it seems to increase over time [41]. Moreover, it can negatively affect the properties of orthodontic archwires [42].

Bacteria adapted to sub-inhibitory concentrations of nickel, 62.5–250 μg/mL, were more resistant to some antimicrobial agents. Strains adapted to 250 μg/mL showed the highest adhesion potential and had a tendency to higher resistance for five tested antibiotics. It might be that the changes of bacteria-induced by nickel contamination influenced both phenomena. It was shown that bacteria in an environment with the presence of nickel become resistant to antibiotics [43,44,45]. In addition, the sub-inhibitory concentrations of metal seem to be the ones to provoke the occurrence of resistance [46]. Perhaps small concentrations of metals activate the bacterial defense mechanisms in the form of inducing resistance to antimicrobial agents, which also affects the polarity of the membrane, so they are hydrophobic and less adherent to wet metal wires and less weakly bound within the biofilm. Nevertheless, the difference is only visible at the beginning, on adhesion and early biofilm. Bacteria phenotypically change when they form a biofilm and become protected in a biofilm mass. So far, it is not known whether commensal bacteria in patients wearing intraoral orthodontic appliances could demonstrate some differences in that area. Results of this study imply that an effect on bacteria could be possible. Bacteria that were subjected and adapted to higher doses, 500–1000 μg/mL, were more sensitive to antibiotics. It is also known that metals are sometimes used as a synergist for antibiotics, so these findings are not surprising [47]. Even so, these higher concentrations are unlikely to be found in the oral cavity. Nickel, in large doses, has a negative effect on the human body, but the use of NiTi-alloys in orthodontic appliances is acceptable since it is temporary and not implanted forever.

The limitation of this study was the in vitro nature of it. This study reports that establishing the model of exposure and adaptation to Ni ions with *S. aureus*. Only one type of bacteria was used in the experiments and Gram-positive bacteria are known to be more susceptible to antibacterial agents. Further research should include comparative studies with the use of Gram-negative bacteria that are more resistant to antibiotic therapy. With some tendencies demonstrated, further research should also be aimed at assessing the bacteria collected from saliva samples or biofilm and testing their nickel accumulation and sensitivity to antimicrobial agents, including oral antiseptics and mouthrinses.

## 5. Conclusions

Bacteria adapted to nickel, adhered slightly better to NiTi wires than the non-adapted bacteria. Biofilm formation was also higher in strains adapted to lower concentrations of nickel. Bacteria adapted to lower concentrations of nickel demonstrated more resistance to antimicrobial agents than the control strain. Those adapted to higher nickel concentrations were more susceptible to antimicrobial agents and displayed reduced adhesion.

## Data Availability

The data presented in this study are available on request from the corresponding author.
